# Hibernoma: a case report of a rare cardiac tumour

**DOI:** 10.1093/ehjcr/ytad612

**Published:** 2023-12-26

**Authors:** David Cistulli, Farrah Othman, Rooshdiya Karim, Rajesh Puranik

**Affiliations:** Royal Prince Alfred Hospital, 50 Missenden Road, Camperdown, NSW, Australia; Royal Prince Alfred Hospital, 50 Missenden Road, Camperdown, NSW, Australia; Royal Prince Alfred Hospital, 50 Missenden Road, Camperdown, NSW, Australia; New South Wales Health Pathology, Australia; University of Sydney, Camperdown, NSW, Australia; Royal Prince Alfred Hospital, 50 Missenden Road, Camperdown, NSW, Australia; University of Sydney, Camperdown, NSW, Australia

**Keywords:** Hibernoma, Lipoma, Cardiac tumour, Cardiac biopsy, Case report

## Abstract

**Background:**

A cardiac hibernoma is a rare phenomenon, with just a handful of reports in the literature. They are difficult to characterize with conventional imaging including echocardiography, computed tomography (CT), cardiac magnetic resonance (CMR), or positron emission tomography (PET). Their definitive diagnosis relies primarily on histopathology via either endovascular or surgical biopsy. Previous case reports have entailed surgical excision followed by histopathology; however, surgery may be unfavourable in some patients with increased perioperative risk.

**Case summary:**

We present the case of a 57-year-old woman who was referred to our cardiology service with an interatrial lipomatous mass found incidentally on chest CT for assessment of rib fractures. She had 6 months of unexplained syncope, which was attributed to superior vena cava (SVC) compression demonstrated by chest CT. The mass had benign characteristics on echocardiography, CT, and CMR but was glucose-avid on PET, which indicated a possible malignancy such as liposarcoma. Her comorbid and very significant airways disease precluded her from surgical excision, so instead, endovascular biopsy was performed. Histopathology showed brown fat which was negative for mouse double minute 2 amplification on fluorescence *in situ* hybridisation testing; hence, a diagnosis was made of hibernoma, a rare benign tumour of brown fat. Given the benign diagnosis and her surgical risk with severe chronic obstructive pulmonary disease, a multidisciplinary recommendation was made favouring conservative management, with careful ongoing follow-up and the consideration of SVC stenting if symptoms progressed.

**Discussion:**

The definitive diagnosis of a cardiac hibernoma is complex and relies heavily on histopathology due to the contradictory findings on chest imaging. Careful consideration of management within a multidisciplinary team setting is essential to achieve a successful outcome.

**Learning points**

- Although multimodal imaging including echocardiography, cardiac magnetic resonance (CMR), and positron emission tomography (PET) are generally useful for guiding the diagnosis and management of most intracardiac masses, lipomatous intracardiac masses can produce contradictory findings.
- Hence, histopathology, in particular the presence of mouse double minute 2 (MDM2), is important for confirming the diagnosis of liposarcoma. Conversely, the absence of MDM2 is more suggestive of a benign lipoma or hibernoma.
- Non-operative management of cardiac hibernoma is a potentially viable option for patients with unacceptably high surgical risk, where CMR has significant utility for monitoring and surveillance.

## Introduction

The diagnostic approach and management of cardiac tumours is challenging, particularly those which demonstrate lipomatous features on imaging.^1–10^ This case describes a cardiac hibernoma, which is a rare benign brown adipose tumour described in just a handful of case reports.^1–10^ Diagnosis of hibernoma is challenging with conventional chest imaging, with all previous case reports relying heavily on post-excisional biopsy to confirm the diagnosis.

There are no reported guidelines on the management of cardiac lipomas, let alone hibernomas.^1^ In the scarce case reports, surgical resection was universally performed due to the risk for lipomas to grow or even infiltrate/compress nearby structures.^1^ In this case, a multidisciplinary decision was made for non-operative management given concurrent comorbid severe airways disease, which represents the first cardiac hibernoma, to our knowledge, managed conservatively.

## Summary figure

**Figure:**
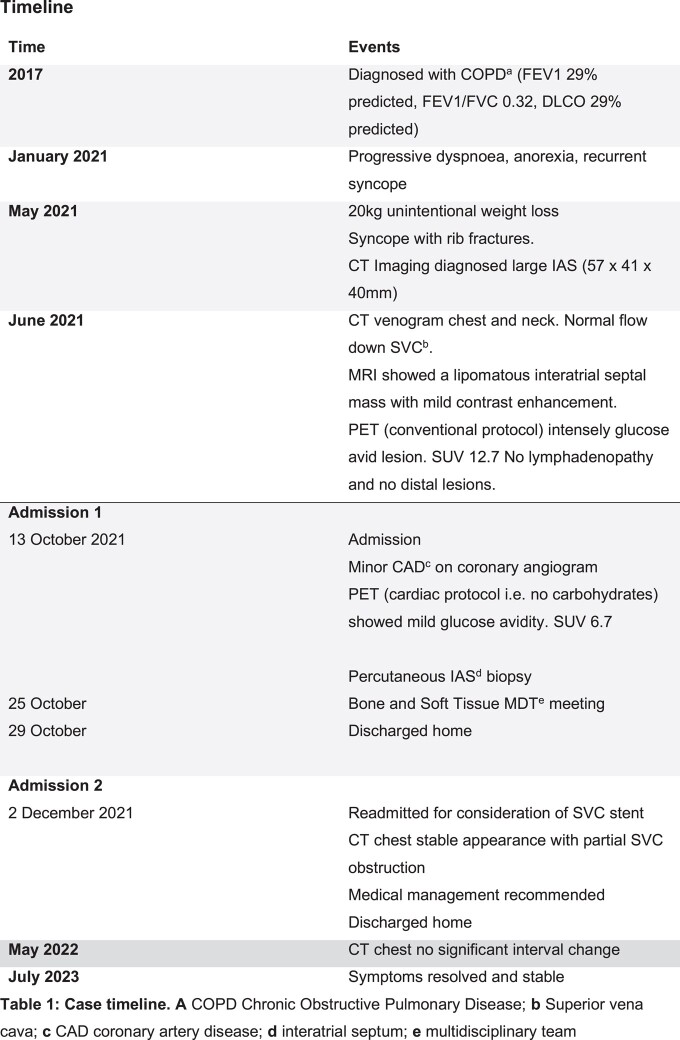


## Case presentation

A 57-year-old female of Indigenous Australian descent was referred to our cardiology service for evaluation of a large interatrial mass incidentally identified on chest imaging performed for assessment of rib fractures, occurring in the context of unexplained syncope. She is an ex-smoker (cigarettes, 28 pack/years) and used marijuana daily. Both were stopped 6 months before presentation and resulted in severe chronic obstructive airways disease (FEV1 0.74 L, 29% predicted; FEV1/FVC 0.32; DLCO 29% predicted). Her only other previous medical history was of Caesarean procedure and depression. Her regular medications were duloxetine, and the following inhalers: budesonide–formoterol, tiotropium, and salbutamol as required.

The patient described 6 months of multiple syncopal episodes. These occurred after periods of recumbency, and there were no symptoms preceding these episodes. An invasive coronary angiogram demonstrated minor coronary disease, while echocardiography showed normal bi-ventricular function and telemetry ruled out significant rhythm disturbances.

A computed tomography (CT) chest without contrast performed May 2021 reported rib fractures as was clinically suspected, but also superior vena cava (SVC) obstruction from a 57 × 41 × 40 mm predominantly fat signal mass. A whole-body positron emission tomography (PET) performed at her local centre demonstrated marked avidity for fluorodeoxyglucose (FDG) by a fat density mass in the interatrial septum (IAS), most consistent with a malignant liposarcoma. There was no lymphadenopathy or distant metastases. She was subsequently referred to our centre for further investigations and management.

On presentation to our centre, she was 82 kg, blood pressure 125/87 mmHg, and heart rate was 92 b.p.m. in an ectopic atrial rhythm on electrocardiogram (ECG). There was no right heart strain. Oxygen saturations were between 90% and 98% on room air throughout the entire admission. She had markedly hyperexpanded lungs and no clinical features of pulmonary hypertension. There were no palpable lymph nodes. She had mildly positive Pemberton’s sign.

**Figure 1:**
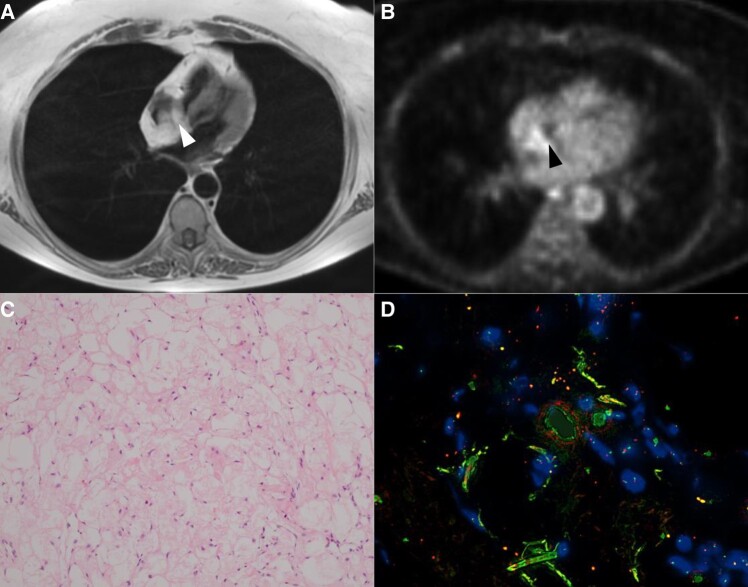
Imaging and histopathology. (*A*) Cardiac magnetic resonance demonstrating lobulated interatrial mass (white arrow); (*B*) Positron emission tomography scan demonstrating moderate fluorodeoxyglucose (FDG) uptake within the interatrial septum (black arrow). The scan was performed 60 min post intravenous administration of 282 MBq of FDG; (*C*) Haematoxylin and eosin stain demonstrating brown adipose and native mature adipose tissue; (*D*) Fluorescent *in situ* hybridisation demonstrating normal signal without amplification of mouse double minute 2 locus.

Echocardiography at our centre identified the IAS and mass in both transthoracic and transoesophageal views (see Supplementary material online, *Videos S1 and S2*, respectively). The patient underwent a CT venogram of the chest and neck, which demonstrated normal flow down the SVC. She then proceeded to have dedicated cardiac magnetic resonance (CMR) imaging to further delineate the IAS and mass. *Figure 1A* demonstrates the interatrial septal mass was lobulated, extending posteriorly and superiorly into the pericardial space. On black blood and cine imaging, it was markedly hyperintense and hypointense on fat-saturated T2-weighted imaging, relative to the myocardium. There was mild enhancement noted post administration of gadolinium contrast. These signal characteristics were more consistent with a benign process.

As shown in *Figure 1B*, repeat PET using a cardiac dedicated protocol (i.e. avoiding pre-scan carbohydrate) found only moderately increased metabolism compared with the previous PET imaging (SUV 6.5 vs. 12.7). Importantly, there was no brown fat uptake; however, given moderate metabolism shown in the mass, our cardiothoracic surgeons were consulted for feasibility of biopsy. As she was too high risk for surgical management and biopsy, a multidisciplinary team (MDT) discussion determined that biopsy would be crucial to ascertain whether the mass was benign or malignant. The mass was deemed not anatomically feasible for CT-guided biopsy, and hence, we proceeded to a transoesophageal echocardiogram (TOE) guided endovascular biopsy of the IAS mass.

The biopsy result demonstrated two fragments of cardiac muscle and one of a fatty process that included brown fat and mature appearing adipocytes, as seen in *Figure 1C*. There was no lymphoid process identified nor was there amyloid or iron deposition seen on Congo Red and Perls staining, respectively. *Figure 1D* demonstrates that fluorescence *in situ* hybridisation testing (FISH) testing for mouse double minute 2 (MDM2) was negative (sensitivity of about 99% in atypical lipomatous tumours or well-differentiated and de-differentiated liposarcoma).^11^ We hence conclude that the lesion is a hibernoma based on morphology and negative MDM2 FISH.

She was discharged home with a plan for serial surveillance imaging and telehealth to accommodate her regional residence. Aspirin 100 mg daily was added to her medications to reduce her risk of thrombotic events. A progress CT chest in May 2022 demonstrated no significant interval changes of the mass, while a telephone interview in July 2023 found she had no further syncopal episodes, especially after following advice on adequate levels of oral intake and hydration. She may be considered for SVC stenting if her symptoms progress.

## Discussion

This case presents an uncommon cardiac tumour in which there are both difficulties in discerning the diagnosis and the optimal management approach.

A hibernoma is a rare benign brown adipose tumour, which typically occurs in the subcutaneous tissue of the limbs, neck, and trunk.^1–3^ It is uncommon to originate in the heart, with few past reports in the literature.^1–10^ A recent systematic review by Shu *et al.*^1^ described 255 case reports of cardiac lipomas, with only 4 hibernoma. They may be detected incidentally or present clinically with obstructive symptoms.^1–10^ In the very few reported cases, they mostly occur in the IAS or right atrium.^4–10^

Diagnosis of hibernoma is challenging with conventional chest imaging be it echocardiography, CT, CMR, or PET. Based on this case and previous reports, hibernomas are most often well-demarcated lesions with heterogeneous characteristics. Positron emission tomography is especially convoluting because a cardiac hibernoma will be glucose-avid, which is classically an indicator of malignancy.^12–14^ Interestingly, previous imaging studies of brown adipose tissue have found methods of reducing its uptake of glucose, such as keeping the patient warm, using a high-fat/low-carb diet, and administering agents like propranolol or fentanyl.^12^ Our case demonstrates the importance of appropriate pre-PET scan preparation with a low-carbohydrate diet to avoid the possibility of false-positive findings.

Histological findings of a hibernoma are similar regardless of location.^2,3^ It entails a predominance of multivacuolated cells with eosinophilic cytoplasm and small central nuclei, which represents the brown adipocytes.^2,3^ These have a significant vascular supply, much like physiological brown adipose tissue, as well as native cell infiltrates.^4–10^ Hibernoma-like areas can be seen in liposarcomas, hence the importance of MDM2 FISH testing.^3^ All previous case reports have relied heavily on open biopsy to confirm a diagnosis of cardiac hibernoma.

The seemingly contradictory findings in imaging modalities of this case created a significant diagnostic dilemma. The mass had benign characteristics on echocardiogram, chest CT, and CMR, which were suggestive of a lipoma. However, the glucose-avid findings of PET imaging raised suspicion of malignancy such as liposarcoma. Ultimately, biopsy proved to be the most important investigation for diagnosis, with the absence of MDM2 markers ruling out malignant sarcomatous disease.

There are no reported guidelines on the management of cardiac lipomas, let alone hibernomas.^1^ Shu *et al.*^1^ suggest consideration of all patients for surgical resection because of the potential for lipomas to grow and compress nearby structures. They also suggest close follow-up with imaging to monitor growth or recurrence. These recommendations are reciprocated in the scarce case reports on hibernoma, whereby the lesion was always surgically excised.^4–10^

Previous case reports have performed surgical management with a curative intention, the rationale being to prevent progression and obstructive or compressive effects.^4–11^ Currently, there are insufficient past cases of cardiac hibernoma to provide a thorough understanding of natural history. Fatal pulmonary embolism has been reported in an elderly woman,^10^ as well as fatal cardiogenic shock in a neonate,^5^ both of which were diagnosed post-mortem. More broadly, cardiac lipomas may be asymptomatic or present with symptoms of chest pain, dyspnoea, and palpitations to severe rare adverse events including syncope or sudden death secondary to arrhythmia.^1^ Surgical management should be considered carefully on a case-by-case basis due to the theoretical risk of continued slow growth and mass effect leading to complications.^1^ Importantly, there are no reports from the limited reported cases of malignant transformation for hibernoma.^1,2,4–10^ In our case, the patient’s significant airways disease was a contraindication to surgical management; hence, a percutaneous biopsy was performed for diagnostic/prognostic purposes.

Cardiac hibernoma is a rare phenomenon. Diagnosis is aided significantly by multimodal imaging and histopathology with negative MDM2 FISH. This case report demonstrates a successful outcome with conservative management.

## Supplementary Material

ytad612_Supplementary_Data

## Data Availability

The data underlying this article cannot be shared publicly to ensure the privacy of the individual who participated in the study. The data will be shared on reasonable request to the corresponding author.

## Lead author biography

David Cistulli completed his BMedSci in 2016, followed by his MD in 2020. In 2023, he is working as a surgical senior resident medical officer at Royal Prince Alfred Hospital, Sydney, Australia. He is currently completing his Masters and has a strong interest in cardiothoracic surgery.

## Supplementary material

**Consent:** The authors confirm that written consent for submission and publication of this case report including images has been obtained from the patient in line with the Committee on Publication Ethics (COPE) guidance.

**Funding:** None declared.
